# Risk Factors for Normal and High-Tension Glaucoma in Poland in Connection with Polymorphisms of the Endothelial Nitric Oxide Synthase Gene

**DOI:** 10.1371/journal.pone.0147540

**Published:** 2016-01-25

**Authors:** Ewa Kosior-Jarecka, Urszula Łukasik, Dominika Wróbel-Dudzińska, Janusz Kocki, Joanna Bartosińska, Agnieszka Witczak, Grażyna Chodorowska, Jerzy Mosiewicz, Tomasz Żarnowski

**Affiliations:** 1 Department of Diagnostics and Microsurgery of Glaucoma, Medical University, Lublin, Poland; 2 Department of Clinical Genetics, Medical University, Lublin, Poland; 3 Department of Dermatology, Venerology and Paediatric Dermatology, Medical University, Lublin, Poland; 4 Department of Internal Diseases, Medical University, Lublin, Poland; Casey Eye Institute, UNITED STATES

## Abstract

**Aim:**

The purpose of this study was to evaluate the influence of polymorphisms of the eNOS gene on the clinical status of patients with normal and high tension glaucoma.

**Methods:**

266 Polish Caucasian patients with primary open angle glaucoma were studied. Of the 266, 156 had normal tension glaucoma (NTG) and 110 high tension glaucoma (HTG). DNA material was isolated from peripheral venous blood using commercial kits. Real-time PCR reaction was used to amplify the promoter site of the endothelial nitric oxide synthase (eNOS) gene, including the single nucleotide polymorphism (SNP) site T-786C and part of the 7^th^ exon of eNOS, including G894T SNP. Genotypes were determined with TaqMan SNP Genotyping Assays.

**Results:**

There were no significant differences in frequencies of the allelic variants of both polymorphisms. In G894T SNP, however, the wild GG form was more common in the HTG group. The SNP of the eNOS gene did not significantly influence the progression rate in either of the groups studied. There were no differences in variants of the eNOS gene regarding the necessity for and success of surgery and the progression of the disease. In the NTG group, no statistical correlation was observed between G894T, T786C polymorphism variants, and risk factors such as optic disc haemorrhages, optic disc notches, and peripapillary atrophy. Mean diastolic and systolic pressure during the day and night were lowest in NTG patients with the CC variant of the T786C polymorphism. No statistical correlation was observed between the G894T and T786C polymorphisms and capillaroscopic examination results.

**Conclusions:**

Genotype frequencies are similar for both the eNOS G894T and T-786C polymorphisms in NTG and HTG patients. These polymorphisms do not correlate with risk factors and do not influence the state of the capillary system in NTG patients. Systolic blood pressure is lower in NTG patients with mutated alleles of both polymorphisms.

## Introduction

Open-angle glaucoma (OAG), the most common type of glaucoma, is characterized by a slow, progressive loss of the retinal nerve fibre layer (RNFL) in combination with visual field changes corresponding to increased excavation of the optic disc [1]. Primary OAG (POAG) appears in the form of high-pressure open angle glaucoma, where intraocular pressure (IOP) is > 21 mmHg, and normal-tension glaucoma (NTG), where IOP by definition falls within and does not exceed a statistically normal range of 21 mmHg at any time [2].

Glaucoma remains a multi-factorial optic neuropathy of unknown aetiology. Elevated IOP is the most important risk factor for the disease [3]. However, in some glaucoma patients, especially with NTG, reduction of IOP does not fully prevent progression of the disease, which indicates that factors other than elevated IOP are involved in its pathogenesis.

Two mechanisms have been postulated as possible causes of POAG: (1) vascular dysfunction that causes ischaemia in the optic nerve, and (2) mechanical dysfunction via cribriform plate compression of the axons. According to vascular theory, reduction of blood flow to the optic nerve head is an important risk factor involved in damage to retinal ganglion cells [4]. An adequate blood supply here would thus depend on the maintenance of a basal vasodilatator tonus in the ocular arteries, which in turn is guaranteed by constant production of nitric oxide (NO). Nitric oxide participates in two additional processes related to glaucoma pathogenesis: it regulates aqueous humour outflow, and it takes part in the apoptosis of retinal ganglion cells [5].

Nitric oxide, which is synthesized in the vascular endothelium through endothelial nitric oxide synthase on the substrate L-arginine, is a potent modulator of vascular tonus inasmuch as it induces vasodilation. The human eNOS gene is highly polymorphic. However, assessment of the influence of eNOS polymorphisms on gene function indicates that two such polymorphisms appear most important. Ghilardi et al. found that the promoter-region polymorphism T-786C may influence gene transcription [6] by causing a 50% decrease of promoter activity and significantly reducing local NO concentrations [7]. This polymorphism has been considered a risk factor for nonarteritic anterior ischemic neuropathy (NAION) [8], coronary spasms [9], and coronary artery disease [10].

The single nucleotide polymorphism G894T is the only one which alters protein structure by substituting glutamate for aspartate at amino acid position 298; it also has the potential to change one or more functional properties of the enzyme directly [11]. Molecular studies have indicated that intact eNOS Asp298 has an enzymatic activity which is equivalent to that of eNOS Glu298 but undergoes selective proteolysis in native cells and tissues, with the result that the steady-state level of active eNOS may be reduced in carriers of this allele. Carriers of eNOS Asp298, particularly if they are exposed to adverse environmental influences on endothelial function, may be at increased risk of developing atherosclerosis and cerebrovascular disease [12]. This polymorphism has been associated with a risk of NAION [8], ischaemic shock [13], coronary artery disease [10], myocardial infarction [14], and coronary spasms [15].

Changes in the enzymatic activity of eNOS caused by genetic polymorphisms may play a role in the pathogenesis of glaucoma. The aim of the present study was to evaluate single nucleotide polymorphisms (SNPs) of the eNOS gene and their influence on the clinical status of patients with normal and high tension glaucoma.

## Material and Methods

The group studied consisted of 266 Polish Caucasian patients with primary open angle glaucoma treated at the Department of Diagnostics and Microsurgery of Glaucoma, Medical University of Lublin, Poland. Written consent was obtained from all patients before their enrolment in the study, which was enclosed to study documents. The study adhered to the tenets of the Declaration of Helsinki and the design was approved by local ethical committee in Medical University of Lublin (approval number 127/12). The group was divided into 2 subgroups: 156 normal-tension glaucoma patients (109 female (F) and 47 male (M); mean age 72), and 110 high-tension glaucoma patients (76F and 34M; mean age 75). Patients with glaucoma met the following inclusion criteria: reproducibly demonstrable glaucomatous visual field damage in either eye in at least three successive perimetric tests, glaucomatous neuroretinal rim loss, and an open angle under gonioscopy. Patients whose IOP was consistently >21 mmHg were diagnosed with HTG. In NTG patients, IOP was consistently <21 mmHg, and the highest ever measured was ≤21 mmHg.

During regular visits, best corrected visual acuity (BCVA), maximum intraocular pressure (IOP), and IOP with Goldmann applanation tonometry were measured and gonioscopic, pachymetric, visual field (VF) (30–2 SITA-fast, Humphrey), slit light biomicroscopic and stereoscopic fundus examinations were carried out. All the measurements of IOP were corrected according to results of central corneal thickness (CCT) measurements obtained during pachymetry. Mean CCT in NTG patients (535,4 microm) did not significantly differ from HTG group (539,4 microm) In normal-tension glaucoma patients, videocapillaroscopic examination of fingers 2 to 5 and 24-hour automated ambulatory blood pressure monitoring were additionally performed. The medical history was recorded regarding glaucoma, other ophthalmic diseases, chronic general disorders, and vascular risk factors (migraine, low blood pressure and cold extremities).

DNA material was isolated from peripheral venous blood using commercial kits (Qiagen, Boston, USA). Real-time PCR reaction was applied to amplify the promoter site of the eNOS gene, including SNP site T-786C (rs 2070744) and part of the 7^th^ exon of eNOS, including the G894T SNP (rs 1799983). Genotypes were determined with TaqMan SNP Genotyping Assays (Applied Biosystems).

Statistic evaluation of the data was performed using Statistica 10. The results were reported mainly as the mean±SD. A p-value of less than 0.05 was considered statistically significant. Normality distribution was checked by Shapiro-Wilk test. For non-normally distributed data Mann-Whitney test was used. Quantitative variables with the normal distribution were analysed by paired Student t-test. When we did not meet the normality requirements therefore, the non-parametric test were used. Proportions were analysed by chi-square test with Yates correction if necessary. For quantitative variables one way analysis of variance ANOVA was used and comparisons post-hoc in the form of Tukey’s test. For multiple regression analysis Pearson’s correlation was used. The observed genotype frequencies for each SNP were tested for Hardy–Weinberg equilibrium (HWE) in both cases and controls and the difference between the observed and expected frequencies was tested for significance using Fisher exact test. Allelic or genotypic frequencies between two studied groups were compared by Chi-2 test. It can be estimated that there are about 420000 adult patients with diagnosed glaucoma in Poland and our study group of 266 Polish glaucoma patients can support confidence interval 6 at the 95% level of confidence.

## Results

### 1. eNOS Polymorphisms in NTG and HTG

The distributions of genotypes and alleles in NTG and HTG groups were consistent with the Hardy-Weinberg equilibrium.

The TT genotype of Glu298Asp was present in 8.4% of the patients with glaucoma in this study; GG was observed in 44.9%, and GT in 46.7%. CC was present in the eNOS gene's T894C polymorphism in 11.8% of the patients, TT in 40.1%, and CT in 48,1%. There were no significant differences in the frequencies of allelic variants of the two polymorphisms after the group was divided according to the respective glaucoma subtype. In G894T SNP, however, the wild GG form was more common in the HTG group (Table 1).

**Table 1:** Frequency of allelic variants of polymorphisms of the eNOS gene in NTG and HTG patients.

**rs 179993**							
	TT	GG	GT	p value	G allele	T allele	p value
**NTG**	13(8.6%)	57(37.5%)	82(53.9%)	0,009	196(64.5%)	108(35.5%)	0,022
**HTG**	8(8.2%)	55(56.7%)	34(35.1%)		144(74.2%)	50(25.8%)	
**rs 2070744**							
	TT		CT	p value	C allele	T allele	p value
**NTG**	56(36.4%)	20(13.0%)	78(50.6%)	0,330	118(38.3%)	190(61.7%)	0,165
**HTG**	49(45.4%)	11(10.2%)	48(44.4%)		70(32.7%)	146(67.3%)	

The groups were divided according to gender, and the frequencies of allelic variants were compared. Although no significant differences were found, the CC variant of the T-786C polymorphism was more than twice as frequent in NTG women as in men, whereas no such difference was found among the HTG patients (Table 2).

**Table 2:** Frequency of allelic variants of polymorphisms of the eNOS gene in female and male patients.

**rs 179993**							
	**TT**	**GG**	**GT**	**p value**	**G allele**	**T allele**	**p value**
**NTG**							
**F**	9(8.6%)	41(39.4%)	55(52.8%)	0.88	73 (34,7%)	137(65,3%)	0.71
**M**	4(8.7%)	16(34.8%)	26(56.5%)		34(37%)	58(63)	
**HTG**							
**F**	5 (7.5%)	39(58.2%)	23(34.3%)	0.89	125(59,5%)	85(40,5%)	0.23
**M**	3(10.3%)	16(55.2%)	10(34.5%)		64(66,6%)	32(33,4%)	
**rs 2070744**							
	**TT**	**CC**	**CT**	**p value**	**C allele**	**T allele**	**p value**
**NTG**							
**F**	37(35.2%)	17(16.2%)	51(48.6%)	0.24	33(24,7%)	101(75,3	0.66
**M**	19(39.6%)	3(6.2%)	26(54.2%)		16(27,6%)	42(72,4%)	
**HTG**							
**F**	30(44.8%)	7(10.4%)	30(44.8%)	0.89	90(67,1%)	44(32,9%)	0.78
**M**	13(50.0%)	3(11.5%)	10(38.5%)		36 (69,2%)	16(30.8%)	

The heterozygotic GT variant of the G894T polymorphism was more common among women in the NTG group than among women in the HTG group. However, this difference did not reach statistical significance. The most frequent genotype of G894T among NTG men was heterozygotic, whereas wild GG was predominant among HTG men. No association was observed between the genotype variants of the two polymorphisms under study (Table 3).

**Table 3:** Association of the polymorphic variants studied in NTG and HTG patients.

		rs 2070744					
rs 1799983		NTG			HTG		
		TT	CC	TC	TT	CC	TC
	TT	0	4(2.7%)	9(6.0%)	0	2(2.2%)	4(4.4%)
	GG	30(20%)	5(3.3%)	21(14%)	32(35.2%)	2(2.2%)	19(20.9%)
	GT	24(16%)	11(7.3%)	46(30.7%)	9(9.9%)	7(7.7%)	16(17.6%)
		p = 0.08			p = 0.1		

### 2. Influence of eNOS polymorphisms on ophthalmic status

#### Initial IOP

As we had expected, there was a significant difference in initial IOP between the NTG and the HTG groups. However, the highest values for G894T were observed in patients with the TT genotype, whereas initial IOP levels were similar for both the wild and heterozygotic variants. Similarly, maximum IOP for T-786C in both groups was highest in patients with the CC genotype. Detailed results are found in Table 4.

**Table 4:** Maximum IOP according to the allelic variants of two polymorphisms.

	IOP (mmHg)					
	rs 1799983			rs 2070744		
	TT	GG	GT	TT	CC	TC
**NTG**	18.4	17.3	17.1	17.3	17.8	17.3
**HTG**	26.7	24.6	24.5	24.1	26.8	25.1
	F = 13.25; p = 0.000000			F = 12.31; p = 0.000000		

#### Visual field examination

In the NTG group, mean deviation (MD) at the time of glaucoma diagnosis was -6,0 dB, whereas in HTG it was significantly greater (-10.52 dB, p = 0.0008*). The mean deviations in both groups are found in Table 5. The observed progression was not related to the polymorphisms under study. Table 6 shows the frequencies of progressive forms of glaucoma in relation to specific genotypes.

**Table 5:** Staging of glaucoma in the two groups according to mean deviation.

MD	0-6db	6-12dB	12-20dB	Over 20dB
**NTG**	51.8%	20.6%	18.4%	9.2%
**HTG**	33.3%	18.7%	24.0%	24.0%
p = 0.0022, OR = 2.42				

**Table 6:** Relation of observed progression to allelic variants of the polymorphisms studied.

**NTG**						
	**rs 1799983**			**rs 2070744**		
	TT	GG	GT	TT	CC	TC
Progression	6(36%)	11 (61%)	33 (77%)	19 (54%)	6 (54%)	28(47%)
**HTG**						
	**rs 1799983**			**rs 2070744**		
	TT	GG	GT	TT	CC	TC
Progression	7 (46%)	10 (40%)	3(50%)	10(29%)	9(45%)	11(22%)

#### Results of antiglaucoma surgery

Primary trabeculectomy was performed in 40 eyes with NTG and 35 eyes with HTG. Although a 30% reduction of initial IOP was observed in 52,4% of NTG eyes and 85.7% of eyes with HTG (Fisher test, RR = 0.,61, 95% Confidence Interval (CI) 0.44–0.85), the planned reduction of IOP did not stop the progression of glaucomatous visual field deterioration in 65% (26 eyes) in NTG patients and 40% (14 eyes) in HTG patients. The likelihood that surgery would slow the progression of IOP was 50% lower in NTG patients than in HTG patients (Fisher test, RR 0,58, 95% CI 0,14–0,91).

The eNOS gene SNP studied here had no significant influence on progression rates in the two groups studied. That is, no differences were found regarding the need and success of surgery or the slowing of progression with regard to the eNOS gene variants under study.

### 3. The Influence of eNOS Polymorphisms on Vascular Risk Factors

Vasoconstrictive factors were detected in 28% of the NTG group and in 2% of the HTG group (Chi-square test, p<0,0001). In the NTG group, optic disk haemorrhages were detected in 14 cases (15%). In the HTG group, optic disk haemorrhages were detected in 1 case (2%) (Chi-square test, p = 0,04). In the NTG group, an optic disk notch was observed in 62 cases (64%). In the HTG group, an optic disc notch was observed in 9 cases (18%) (Chi-square test, p<0,0001).

In the NTG group, no statistical correlation was observed between the G894T and T786C polymorphism variants and risk factors such as optic disc haemorrhages, optic disc notches, and peripapillary atrophy (Chi-square test, p>0,05). The results are presented in Table 7.

**Table 7:** Relation between local glaucoma risk factors and polymorphic variants in NTG patients.

	rs 1799983			rs 2070744		
	GG	GT	TT	TT	TC	CC
**HAEMORRHAGES**	17% (3)	11% (7)	7% (1)	11.4% (4)	13.7% (7)	9% (1)
**NOTCH**	66.7% (12)	65.6% (40)	50% (7)	60% (21)	66% (31)	63.6% (7)
**PPA**	17% (3)	21%(13)	21% (3)	23% (8)	22% (11)	0% (0)

### 4. The Influence of eNOS Polymorphisms on Cardiovascular Status in NTG Patients

#### 24-hour automated ambulatory general blood pressure monitor

Lowest mean diastolic and mean systolic blood pressure values during the day and during the night were recorded in NTG patients with the polymorphic TT variant of the G894T SNP. With regard to diastolic blood pressure at night, the difference was statistically significant.

Mean diastolic and systolic pressure during the day and night were lowest in NTG patients with the CC variant of the T786C polymorphism. The difference regarding mean diastolic BP during the day was significant. Detailed results are found in Table 8.

**Table 8:** Mean blood pressure indices in patients with the allelic variants studied.

	rs 1799983				rs 2070744			
	GG	GT	TT	ANOVA	TT	TC	CC	ANOVA
**Mean systolic BP day (mmHg)**	128.7	133.7	119.3	F = 1.23 p = 0.3	129.6	131.7	129.3	F = 0.09 p = 0.9
**Mean diastolic BP day (mmHg)**	80.3	75.8	67.2	F = 2.95 p = 0.06	78.4	79.9	64.9	F = 7,35 p = 0.002
**Mean systolic BP night (mmHg)**	113.2	110.8	105.7	F = 0.40 p = 0.6	113.3	110.4	106.6	F = 0.49 p = 0.62
**Mean diastolic BP night (mmHg)**	68.6	64.0	53.8	F = 3.43 p = 0.04	66.9	66.4	57.2	F = 2.39 p = 0.1

#### Capillaroscopic examinations

A capillaroscopic examination was performed in 35 NTG patients (23 female and 12 male). 31 of these patients (88.6%) suffered from cold extremities. The patients with NTG presented the following nailfold capillaries: megacapillaries or dilated capillaries (44.4%), ramified/bushy (18.9%), coiled (17.1%).

With regard to the eNOS G894T polymorphism, the GG genotype was present in 6 cases (18%), GT in 24 cases (67%), and TT in 5 cases (15%). With regard to the T786C polymorphism, the TT variant was present in 13 cases (36%), TC in 18 cases (52.6%), and CC in 4 cases (11.3%). No statistical correlation was observed between the G894T and T786C polymorphisms and capillaroscopic examination results.

## Discussion

The frequencies with which allelic variants of the eNOS gene appear in various population groups have been assessed in many studies. However, no such study of glaucoma patients in Poland has been yet published. The TT variant of the G894T polymorphism was found in 8.6% of the NTG patients in this study and in 8.2% of the HTG patients. The data are similar to those found in healthy populations (9.2–9.8%) [16]. However, a more significant distribution is observed in the frequency of the CC variant of the T786C polymorphism. In this study, the CC variant was present in 13% of NTG patients and 10.2% of HTG patients, with the highest frequency in NTG women (15.9%) and the lowest in NTG men (6.4%). According to published data concerning healthy populations, the frequency of the CC variant varies between 1.2% and 18.2% [16].

Although nitric oxide plays a role in the pathogenesis of glaucoma along certain pathways, the possibility of a link between polymorphisms of the eNOS gene, with the risk of POAG, remains a subject of controversy. While some authors have found T-786C polymorphisms in the promoter region to be a risk factor [17–19], other studies have found no such association [20–22].

Liao assessed T-786C polymorphisms in NTG patients and found that the distribution of eNOS repeat alleles in subjects both with and without glaucoma failed to reach statistical significance (p = 0.06). The distribution in subjects with NTG or POAG did not differ significantly [19]. In our study, marginal differences were observed in overall polymorphic variant frequencies.

Normal-tension glaucoma is considered to be more strongly affected by non-IOP factors, particularly vascular factors, than high-tension glaucoma. Factors such as migraine, Raynaud phenomenon, nocturnal hypotension, and a history of hypovolemic shock have been shown to be associated with NTG. Associations between NTG and sleep apnea or reduced cerebrovascular blood flow have also been reported. The high prevalence of vasospastic disorders in NTG patients has led to increased interest in the role of the vascular endothelium in such cases. The endothelium is recognized as being an important functional unit in the regulation of blood flow due to its production of different vasoactive substances such as NO. Although a significant difference in the presence of vasoconstrictive risk factors in NTG and HTG patients was observed in our groups, this was not influenced by the eNOS polymorphisms under study. Similarly, changes in the capillaries of fingers observed in NTG patients were not associated with allelic variants.

Some studies [23,24] have shown that genetic variants of the T-786C polymorphism constitute a risk factor for high tension glaucoma only in women. Logan found an association between promoter region polymorphism and POAG in patients with the history of migraine [20], a disease more prevalent in woman and in patients with normal tension glaucoma. In our study, no significant association of genotypes with gender nor with other vascular risk factors of glaucoma was found. In NTG women, however, the CC variant of the T-786C polymorphism was more than twice as frequent as in men, whereas no similar difference was observed in HTG patients, thus confirming the results of other studies mentioned here.

The cells of the conventional human outflow pathway in the eye generate NO, as shown by the ability of these tissues to convert L-arginine to L-citrulline. Although there is evidence of the expression of all three NOS isozymes in the conventional pathway, the data are conflicting as to whether eNOS or inducible (i)NOS is primarily responsible for NO generation. At the cellular level, NO has been shown to relax the trabecular meshwork (TM) and/or to decrease cell volume; this is consistent with an increased outflow facility. Endothelial NOS overexpression lowers IOP by increasing pressure-dependent drainage with eNOS induction at elevated IOPs, leading to an increased pressure-dependent outflow [25]. The data from our study show that maximum IOP in both normal-tension and high-tension glaucoma is influenced by eNOS gene polymorphisms. However, these data require further confirmation from population studies.

Evidence for the involvement of eNOS single-nucleotide polymorphisms in the development of essential hypertension is limited, though the eNOS Glu298Asp polymorphism appears to influence blood pressure response to exercise. This variant also influences endothelial function, with its effects becoming manifest during the adaptive vascular changes of pregnancy [12].

Pereira et al. carried out a meta-analysis of 53 studies involving a total of 40 413 subjects in order to explore the role of eNOS polymorphisms in hypertensive and normotensive patients. In Asian individuals, Asp allele carriers of the G894T polymorphism displayed an increased risk of hypertension as well as a 2 mmHg increase in both systolic and diastolic blood pressure levels. Furthermore, meta-regression analysis by the same authors indicated that the effect of the Glu298Asp genotype on the risk of hypertension might be dependent on total cholesterol status. No effect of the T-786C variant on hypertension was detected [26]. These data are contrary to ours, which showed a decline in mean blood pressure values in carriers of the weak variants of both polymorphisms. However, no studies assessing eNOS polymorphisms in hypotensive patients have yet been published. Huttunen measured blood pressure in patients with bacteraemia and found that the T allele of the Glu298Asp eNOS gene was a risk factor for hypotension in patients with E. coli bacteraemia but not in bacteraemia caused by a gram-positive organism [27].

Several epidemiological studies have shown that elevated systemic BP is associated with a slight increase in IOP [28]. Population-based studies have identified low perfusion pressure as a risk factor for the development of glaucoma. The Baltimore Eye Survey indicated that individuals with diastolic perfusion pressures lower than 30 mm Hg had a sixfold higher risk of developing the disease than individuals with diastolic perfusion pressures greater than 50 mm Hg [28].

Data from the Early Manifest Glaucoma Trial (EMGT) have established lower systolic perfusion pressure as a new predictor of glaucomatous disease progression, suggesting a 50% increase in risk. When assessing the influence of systolic blood pressure on progression, the authors of that trial found that systolic BP 160 mm Hg was not a risk factor in those with higher baseline IOP but was indeed a risk factor in those with lower IOP (less than 21 mm Hg) [29].

In the present study, blood pressure was automatically monitored for 24 hours in patients with NTG. Although mean BP levels were within the normal range, the lowest mean diastolic and systolic pressures were observed in patients with TT in the G894T polymorphism or CC in the T786C gene variants. Additionally, patients with these polymorphic variants had their highest IOP at the time of the diagnosis. Taken together, these factors could indicate a poorer prognosis regarding glaucoma. Upon evaluating the disc and/or the visual field for progressive changes, however, no association with these polymorphisms was found. In a recently published paper, Kang evaluated a panel of markers in vascular tone-regulating genes in 3108 POAG cases and 3430 controls. Six of eight genes were associated with the POAG code for factors involved in endothelial nitric oxide synthase activity, and three of these six (CAV1, ITPR3, and EDNRB) were also associated with early paracentral loss of visual field [30].

Were a correlation to be found between phenotypic features and their genotypic localization in glaucoma patients with regard to ophthalmic status and the presence of risk factors, this might provide new insights into the pathogenesis of glaucoma. Such a potential genetic biomarker would help to identify patients with an enhanced risk of blindness due to glaucoma. The findings of the present study, which concentrated on genetic backgrounds regarding the vascular status of patients with normal tension glaucoma, show a possible correlation between eNOS SNPs, decreased blood pressure, and elevated maximum IOP. However, this study has some limitations. The main is the relatively small group of patients. That is why obtained results need to be confirmed in the large population study. However, our results have a big advantage of being performed on clinically well-characterized population of a single ethnicity (Caucasian) resident of the small area.

## Conclusions

The eNOS G894T and T-786C polymorphisms appear with similar frequencies in normal tension glaucoma patients and primary open angle glaucoma patients. Endothelial nitric oxide synthase G894T and T-786C polymorphisms do not correlate with NTG risk factors and do not influence the state of the capillary system in NTG patients. Lower systolic blood pressures are present in NTG patients with mutated alleles of both polymorphisms. There may be a correlation between eNOS SNPs, decreased blood pressure, and elevated maximum IOP. Confirmation of this, however, will require further study.
